# A study on use of animals as traditional medicine by Sukuma Tribe of Busega District in North-western Tanzania

**DOI:** 10.1186/s13002-015-0001-y

**Published:** 2015-05-07

**Authors:** Rajeev Vats, Simion Thomas

**Affiliations:** School of Biological Sciences, College of Natural and Mathematical Sciences, the University of Dodoma, Dodoma, Tanzania

**Keywords:** Ethnozoology, Traditional Medicine, Medicinal animals, Tanzania

## Abstract

**Background:**

Faunal resources have played an extensive range of roles in human life from the initial days of recorded history. In addition to their importance, animals have been acknowledged in religion, art, music and literature and several other different cultural manifestations of mankind. Human beings are acquainted with use of animals for foodstuff, cloth, medicine, etc. since ancient times. Huge work has been carried out on ethnobotany and traditional medicine. Animal and their products are also holding medicinal properties that can be exploited for the benefit of human beings like plants. In Tanzania, many tribal communities are spread all over the country and these people are still totally depended on local customary medicinal system for their health care. In the world Tanzania is gifted with wide range of floral and faunal biodiversity. The use of traditional medicine from animals by Sukuma ethnic group of Busega district is the aim of the present study.

**Method:**

In order to collect the information on ethnozoological use about animal and their products predominant among this tribe in Busega district, a study was carried out from August 2012, to July 2013. Data were collected through semi-structured questionnaire and open interview with 180 (118 male and 62 females) selected people. The people from whom the data were collected comprise old age community members, traditional health practicener, fishermen and cultural officers. The name of animal and other ethnozoological information were documented. Pictures and discussion were also recorded with the help of camera and voice recorder.

**Result:**

A total of 42 various animal species were used in nearly 30 different medicinal purposes including STD, stoppage of bleeding, reproductive disorders, asthma, weakness, tuberculosis, cough, paralysis and wound and for other religious beliefs. It has been noticed that animal used by Sukuma tribe, comprise of seventeen mammals, seven birds, four reptiles, eight arthropods and two mollusks. Some of the protected species were also used as important medicinal resources. We also found that cough, tuberculosis, asthma and other respiratory diseases are the utmost cited disease, as such, a number of traditional medicines are available for the treatment.

**Conclusions:**

The present work indicates that 42 animal species were being used to treat nearly 30 different ailments and results show that ethnozoological practices are an important alternative medicinal practice by the Sukuma tribe living in Bungesa district. The present study also indicates the very rich ethnozoological knowledge of these people in relation to traditional medicine. So there is a critical need to properly document to keep a record of the ethnozoological information. We hope that the information generated in this study will be useful for further research in the field of ethnozoology, ethnopharmacology and conservation approach.

## Background

Faunal resources have played a wide range of roles in human life from the earliest days of recorded history. Human beings are familiar with use of animals and plants for food, cloth, medicine, etc. since ancient times [1,2]. The study of relationship between the human societies and the animal resources around them deals under Ethnozoology [3]. Since prehistoric time’s animals, their parts, and products have created part of the inventory of medicinal substances used in numerous cultures [4]. The world health organization estimates that most of the world’s population relies primarily on animal and plant based medicines [5]. Of the 252 indispensible chemicals that have been selected by the World Health Organization, 8.7% derived from animals [6]. In Brazil, Alves *et al.* reported the medicinal use of 283 animal species for the treatment of various ailments [7]. In Bahia state, in the northeast of Brazil, over 180 medicinal animals have been recorded in traditional health care practices [8]. In Traditional Chinese Medicine more than 1500 animal species have been recorded to be some medicinal use [9]. Alves and Rosa recorded the use of 97 animal species as traditional medicine in urban areas of NE and N Brazil [10]. Lev and Amar conducted a survey in the selected markets of Israel and found 20 animal species, which products were sold as traditional drugs [11]. Tamang people of Nepal identify the 11 animal species for used in zootherapeutic purposes [12]. Alves and Rosa in the North and north- east regions of Brazil carried out a survey in fishing communities and recorded 138 animal species, used as traditional medicine [13]. Alves *et al.* also reported nearly 165 reptile’s species were used in traditional folk medicine around the world [14]. Alves conducted a review study in Northeast Brazil and lists 250 animal species for the treatment of diverse ailments [15]. Lev and Amar conducted a study in the selected markets in the kingdom of Jordan and identified 30 animal species, and their products were retailed as traditional medications [16]. In India use of traditional medicine are documented in works like Ayurveda and Charaka Samhita. A number of animals are mentioned in Ayurvedic system, which includes 41 Mammals, 41 Aves, 16 Reptiles, 21 Fishes and 24 Insects [17]. Different ethnic group and tribal people use animals and their products for healing practices of human ailments in present times in India [18]. In Hindu religion people used the various products obtained from the cow viz. milk, urine, dung, curd and ghee since ancient times [19].

Tanzania is gifted with immense faunal and floral biodiversity, because of the thrilling variation in geographical and climatic condition prevailing in the country. In Tanzania, traditional medicine has existed even before colonial times. It used to play a vital role in the doctrine of chiefdoms that existed during pre-colonial era. Colonialists, with their intension to rule Africa had to find a way to discourage all sort of activities which would have provided an opportunity for developing Africans [20]. In Tanzania, different tribal communities are dispersed all over the country, people of these communities are extremely knowledgeable about the animals and their medicinal value, and they also deliver extensive information about the use of animals and their by-products as medicine. Most of the tribal people are totally dependent on local traditional medicinal system for their health care because they are living in very remote areas where hospital and other modern medicinal facilities are not available and even negligible, so they use their traditional knowledge for medicinal purpose and this knowledge is passed through oral communication from generation to generation. It is estimated that more than 80% of the rural population in Tanzania depends on the traditional medicine [21].

A lot of work has been done on utilization of plants and their products as traditional and allopathic medicine in the world. Like plants, animal and their products also keep medicinal properties [22]. Most ethnobiological studies conducted in Tanzania have focused on traditional knowledge of plants and less in animals [23,24]. A little work has been done in Ethnozoology in Tanzania and particularly no work is documented in Sukuma tribe and there is a definite scarcity of ethnobiological knowledge when it comes to animal products. The present study briefly reports an ethnomedicinal/traditional medicinal study among Sukuma tribe in Bugusa district in Tanzania.

## Methods

### The study area

The intended study was carried out in Busega District at Simiyu region. The Busega district is one of five districts in Simiyu Region of Tanzania, namely, Meatu, Itilima, Bariadi, Maswa and Busega. Busega district is located on the northwestern part of Simiyu Region and shares borders with Magu districts in west, Bariadi districts in south, The southeastern part is covered by the Serengeti game reserve and Bunda district. In north side it bordered with Lake Victoria. As a result, many community members utilize both aquatic and terrestrial organisms as a source of medicine.

**Figure 1:**
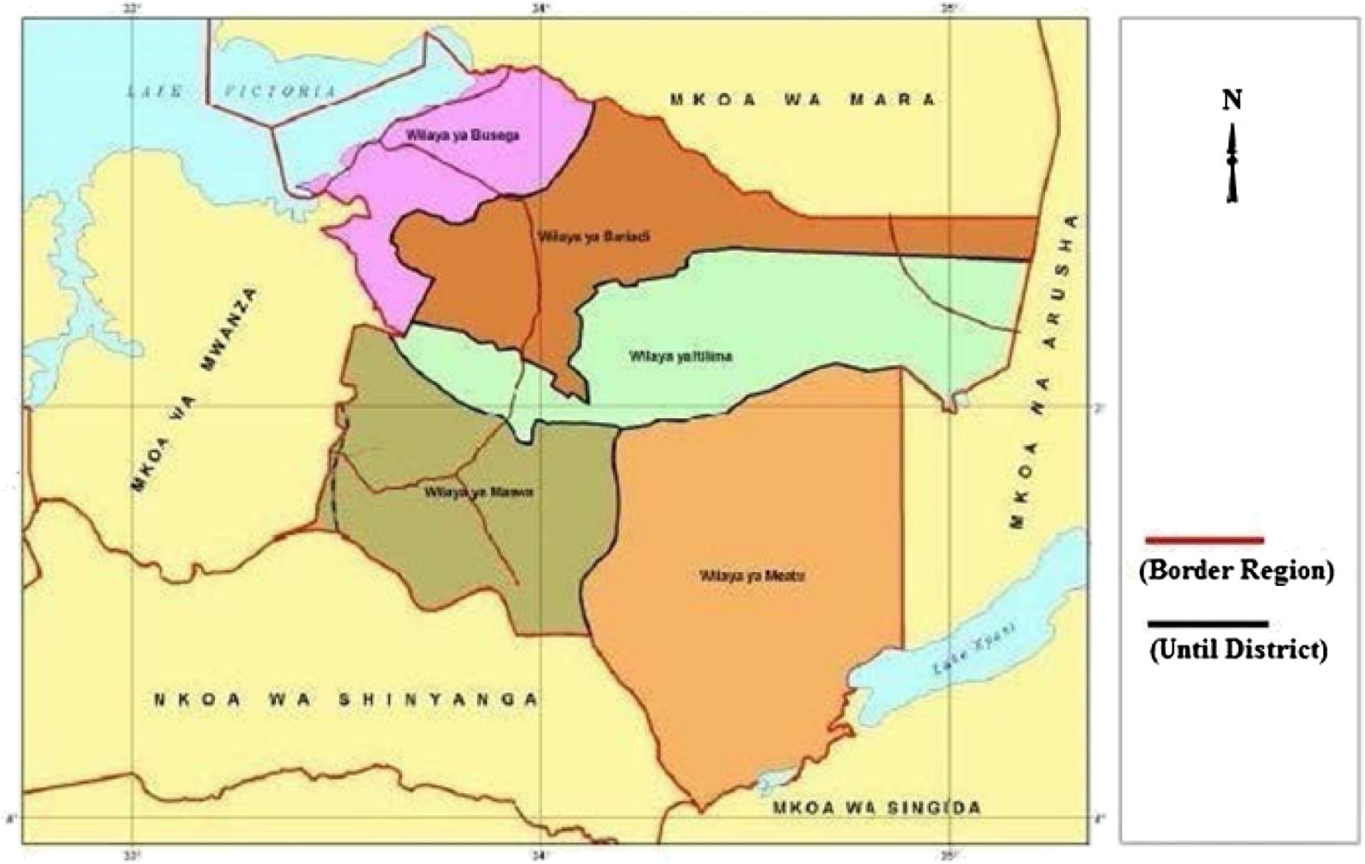
Map of Simiyu region showing all district under the region including District Busega (Wilaya ya Busega).

Busega district is located between latitude 2^0^ 10’ and 2^0^ 50’ South and between longitude 33^0^ and 34^0^ East. The district headquarter is in Nyashimo town. The district is divided into thirteen (13) wards and fifty four (54) villages as per Tanzania Population and Housing Census 2012 [25]. Busega district is Tropical in nature with sun overhead of equator on March and October. Temperature is tropical and range between 25°C and 30°C with average annual temperature of 27°C. There are two wet seasons, the long rains from mid-March to early June, during which the precipitation is between 700 mm to 1000 mm and averages 800 mm per annum and short rains from October to December, during which the rainfall is between 400 mm to 500 mm [26]. Figure 1: Map of the study area.

### The Sukuma tribe

The Sukuma are a patrilineal society; the role of the women being to take care of their husbands and children while men are overseer of the family [27,28]. Young people marry only when they are ready to carry the responsibilities marriage entails. They are initiated into adulthood in a ceremony known as “*lhane”*. The Sukuma do not practice circumcision as part of initiation, but organize a separate ceremony. The young people involved in “*lhane*” have to be prepared well. Respected elders of the community tutor the initiates on their roles and responsibilities in the family and the whole community. The initiates have to think, act and participate as adults in all rituals. After “*lhane*” the initiates are considered adults and cannot be asked to deliver messages anywhere as this is a job for non-initiates [28].

**Figure 2:**
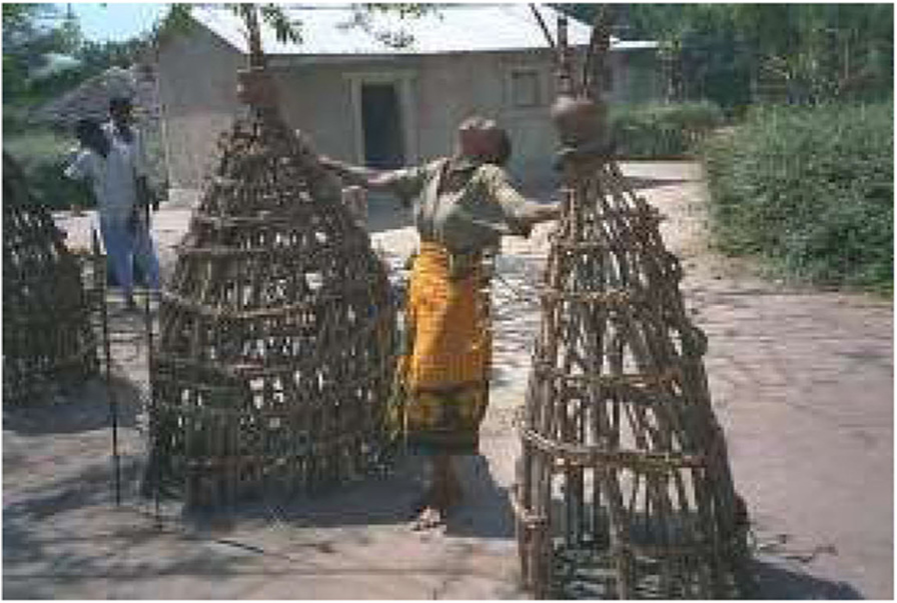
Sukuma lady doing traditional prayer.

**Figure 3:**
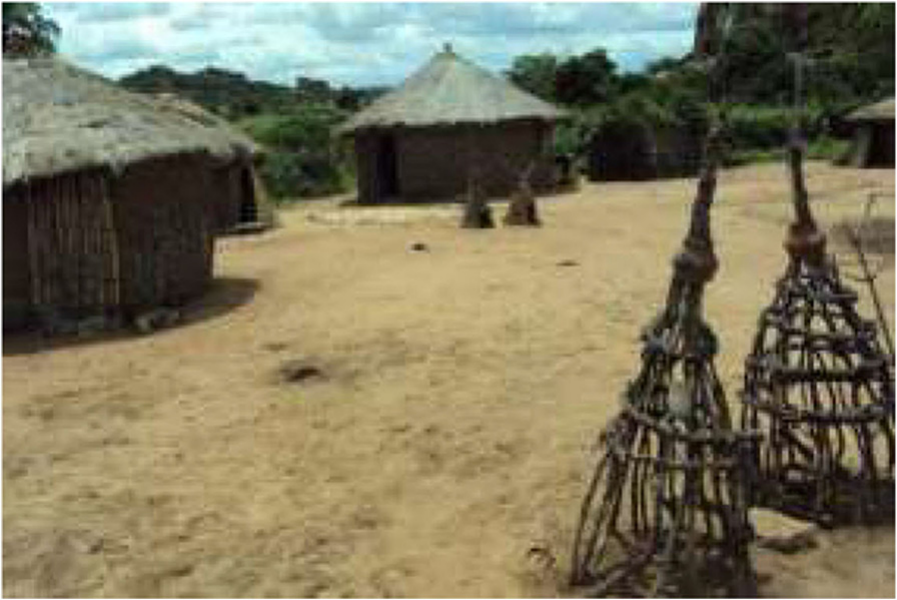
Ancestral shrines in a rural Sukuma healer’s compound.

**Figure 4:**
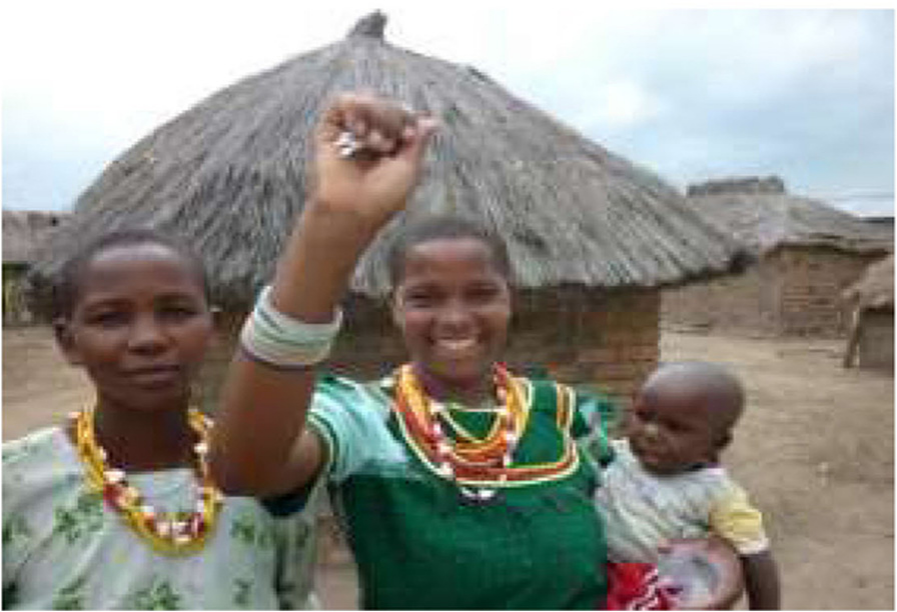
Sukuma lady with her children and traditional house.

**Figure 5:**
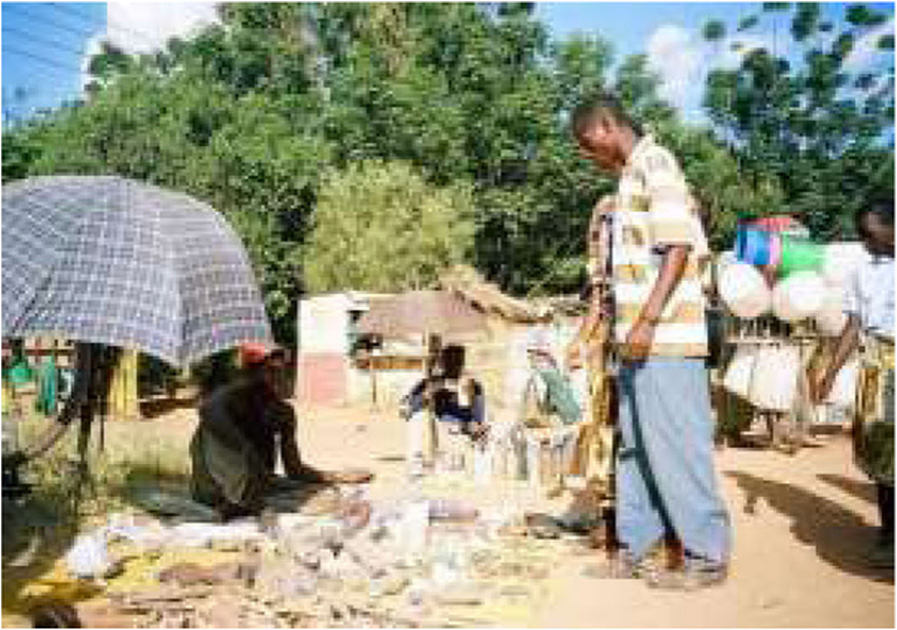
Traditional healers selling medicines in local market.

The Sukuma are believed to being very superstitious, and most will seek aid from the “*Bafumu”*, “*Balaguzi”* and “*Basomboji”* locally used to refer as medicine men, diviners and sooth sayers, respectively. The *Basukuma* have many stories based on their beliefs on death and sufferings. Traditional healers believe that fate is determined by “*Shing’wengwe”* and “*Shishieg’we”*, that is ogres and spirits. The ogres are usually shown as being half human, half demon, or as terrible monsters [28]. The economic condition of the Sukuma people is not good. Agriculture, animal husbandry; poultry forming and laboring are source of income. Educational level is also found very low. The life of the people are full of traditions and social customs from birth to death owning to outdated customs, not attuned to remain competitive in the current economic scenario of privatization [Figures 2, 3, 4, 5].

### Procedures

In order to obtain ethnozoological information about animal and their products used in traditional medicine, a study was conducted from August 2012 to July 2013 in the Busega district of Simiyu region, Tanzania. The ethnomedicinal data (local name of animals, mode of preparation and administration) were collected through semi-structured questionnaire (in their local language mainly Kiswahili, with the help of local mediator), interview and group discussion with selected people of the tribe. The selection of informants was based on their experience, recognition as expert and knowledge old aged person concerning traditional medicine. A total of 180 (118 male and 62 female) people were selected to collect ethnozoological information, these information were collected from local traditional healers, farmers, fisherman and cultural officer. We interviewed 98 (55%) informants within age group 55 and above, followed by 42 informants (23%) with 45 to 54 age group and 40 (22%) with 35–44 years age group.

They were inquired, about the illnesses cured by animal based medicines and the manner in which the medicines were prepared and administered. They were also requested thorough information about mode of preparation and blending of animal products used as ingredients and whether they use animal in the healing practice, since this type of information indicate how a given medicine can be therapeutically effective in term of the right ingredients, the proper dose and the right length of medication. The name of animals and other related information to this study were documented. Some pictures of Sukuma people at their local place and in their life style in study area were taken.

As stated by them, their traditional ethnozoological acquaintance was mainly attained through parental heritage and experience about medicinal value of animal to heal their families or themselves. The scientific name and species of animals were identified using relevant and standard literature [29,30].

### Data analysis

For the data analysis, fidelity level (FL) calculated that demonstrates the percentage of respondents claiming the use of a certain animal species for the same illnesses, was calculated for the most frequently reported diseases or ailments as:

$$ \mathrm{F}\mathrm{L}\left(\%\right) = \mathrm{N}\mathrm{p} \times 100\ /\ \mathrm{N} $$

Where Np is the number of respondents that claim a use of a species to treat a specific disease, and N is the number of respondents that use the animals as a medicine to treat any given disease [31]. The range of fidelity level (FL) is from 1% to 100%. High use value (close to 100%) show that this particular animal species are used by large number of people while a low value show that the respondents disagree on that spices to be used in the treatment of ailments.

## Result and discussion

The present study revealed the traditional medicinal knowledge of treating many types of ailments using different animal and their products by the local Sukuma people inhabitants of Simuyu region, Tanzania. Many old generation people were found to lack formal education, but they have acquaintance about use of local faunal and floral resources for traditional medicinal and other purposes [12], Sukuma people are one of them [Table 1].

**Table 1:** **Knowledge of animal resource use among Sukuma Tribe of Busega District**

**Scientific name**	**Common name (E)**	**Local name (S)**	**Vernacu lar name**	**Parts used**	**Traditional Uses**	**Mode of Preparation**	**Dosage**	**Resp onde nt**	**Use value**	**Conserv ation status**
Mammals										
*Eudorcas thomsonii* (Gunther, 1884)	Thomson’s Gazelle	Nyamela	mbushi	Heart Skin Tail	Treat: asthma, Pneumonia Make drums	Dry, grind pour hot water	Inhale the smoke 1/day*4 days	145	0.80	Status: NT Trend: D
					Chase away insect	Mount flesh skin container				
						Tail is being dried and used				
*Hippopotamu s amphibious* (Linnaeus, 1778)	Hippopotamus	Kiboko	ngubho	Blood	Boost CD_4_ for HIV patient	Blood dried for 3 days	3 spoons/day*	88	0.48	Status: VU
							30 days			Trend: D
*Equus quagga* (Boddaert, 1785)	Plains Zebra	Pundamilia	ndolo	Hooves	Treat: glands	Burn, grind, mix with water	2 cup/day*7 days	122	0.68	Status: LC
										Trend: S
*Atherurus africanus* (Gray, 1842)	Porcupine	Nungunungu	Nungu	Spines	Treat: abscess	Rub ashes in abscess	2/day *2 days	129	0.72	Status: LC Trend: U
*Crocuta crocuta* (Erxleben, 1777)	Spotted Hyena	Fisi	Mbiti	Meat Skin and Feaces	Treat :TB	Eat dry meat Cham	3 pieces/day*3 days.	142	0.79	Status: LC
					For protection		Tie on waist			Trend: D
*Ovis aries* (Linnaeus, 1778)	red Maasai sheep	Kondoo	Ng’oro	Fat	Treat: burn	Extract tail fat	Rub everyday	105	0.58	Status: NA
										Trend: U
*Diceros bicornis* (Linnaeus, 1778)	Black Rhinoceros	Faru	Mhela	Horn	Treat: asthma, gastritis; TB	Paste the horn mix with hot	2/ day* 30 days	96	0.53	Status: CR
										Trend: I
*Phataginus tricuspis* (Rafinesque, 1821)	African Pangolin	Kakakuona	Murhuka ge	Scales	Goodluck	Make charms.	Tie on hand	154	0.85	Status: NT
										Trend: D
*Atelerix albiventris* (Wagner, 1841)	Four-toed Hedgehog	Kalunguyeye	Kilungu miyo	Skin; spines	Stop blood discharge via nostril	Burn; inhale its smoke	Time of suffering	103	0.57	Status: LC
										Trend: S
*Loxodonta Africana* (Blumenbach, 1797)	African Elephant	Tembo	Mhole	Skin	Treat: hepatitis	Burn; get ashes	3 spoon/day*7 days	2	.17	Status: VU
										Trend: I
*Mungos mungo* (Gmelin, 1788)	Banded Mongoose	Nguchiro	Ng’ara	Nail	Treat: cough	Grind and smell	2/day	5	.13	Status: LC
										Trend: S
*Procavia capensis* (Pallas, 1766)	Rock Hyrax	Pimbi	Membe	Urine	Treat: Syphilis	Collect hyrax urinated soil; mix water; filter soil and then drink	1 cup/day*7 days	4	.3	Status: LC
										Trend: U
*Rattus norvegicus* (Berkenhout, 1769)	Brown Rat	Panya	Kitakilan zela	Whole animal	Protection of thieves	Dry the dead rat. and.	embed on farms center	48	.82	Status: LC
										Trend: S
*Kerivoula Africana* (Gray, 1842)	Tanzanian Woolly Bat	Popo	Tunge	Whole animal	Treat : pneumonia	Burn and inhale the smoke	1/day*3 days	7	.37	Status: EN
										Trend: D
*Panthera leo* (Linnaeus, 1778)	Lion	Simba	Shamba	Adipose tissue Skin	Treat ear pus For protection	Rub fat on the ears Make charm	1/day *4 days Tie on neck	11	.62	Status: VU
										Trend: D
*Phacochoeru s africanus* (Gmelin, 1788)	Warthog	Ngiri	Ngere	Tusks	Treat stomach ulcers	Grind, mix with hot water	2 cup/day *7 days	2	.28	Status: LC
										Trend: S
*Lepus capensis* (Linnaeus, 1778)	Cape Hare	Sungura	Sayayi	Fur	For wound healing	Take the fur burn it and	Rub ashes in the wound.	8	.48	Status: LC.
										Trend: D
Insect										
*Aglais urticae* (Linnaeus, 1778)	Butterfly	Kipepeo	Parapapu	Wings	Treat: chest pain.	Grind; Swallow powder	3/day*5 days.	5	.25	Status: NA
										Trend: U
*Lasius niger* (Linnaeus, 1778)	Black ants	Chungu	Sungwa	Whole organism.	To become intelligent and leader	Take the fore ant, grind and rub on head	1/day*3 days	29	.72	Status:
										LC Trend: S
*Butastur rufipennis* (Sundevall, 1851)	Grasshopper Buzzard	Panzi	Ng’umbe	Whole organism	Treat: stomachache; heartbeat	Burn, grind it into powdery form.	Rub 2/day*3 days	54	.86	S tatus: LC
										Trend: D
*Apis mellifera* (Linnaeus, 1778)	Honey bee	Nyuki	Nzoke	Honey	Treat: burn	Rub the burn	2/day*3 days	38	.76	Status: NA
										Trend: U
	Beetle	Kalilila	Kombam wiko	Whole organism	Call a person to come back home	Burn beetle and call the name of a person.	3/day*3 days	48	.82	Status: NA
										Trend: U
**Chilopoda**										
*Scutigera coleoptrata* (Linnaeus, 1778)	Millipede	Tandu		Whole	Treat Dandruff	Burn and swallow the ashes.	1/day*3 days	45	.25	Status: NA
										Trend: U
**Arachnida**										
*Araneus spp* (Clerck, 1757)	Spider	Buibui		Spider web	Stop bleeding.	Apply direct on fresh wound.	Once/ day	99	.55	Status: LC
										Trend: S
**Diplopoda**										
*Trigoniulus corallines* (Gervais, 1847)	Millipede	Jongoo	Igongoli	Whole body	Treat dandruff	Press plasma fluid and swallow	2/day*2 days	2	.51	Status: NA
										Trend: U
**Reptiles**										
*Naja siamensis* (Laurenti, 1768)	Cobra	Cobra	Kipele	Skin	Treat: burns fractured bone	Powder the skin, mixed with water	Rub 2/day*3 days	129	.72	Status: VU
										Trend: D
*Agama mwanzae* (Loveridge, 1923)	Flat-headed Rock Agama	Mjusi	Madhore	Bile	Treat dysentery.	Drink flesh bile	1 spoon/day*3 days	8	.43	Status: LC
										Trend:S
*Python regius* (Shaw, 1802)	Royal Python	Chatu	Nsato	Feaces	Treat back pain	Mix with little water	Rub on back 2/day*3	23	.68	Status: LC
										Trend: U
*Crocodylus niloticus* (Laurenti, 1768)	Nile Crocodile	Mamba	Ng’wina	Skin	Treat TB: gastritis.	Burn and swallow the ashes	2/day*7 days	78	0.43	Status: LC
										Trend: S
**Aves**										
*Baleara reguloum* (Bennett, 1834)	Grey Crowned crane	Korongo	Izunya	blood	Treat stomach ulcers	Drink flesh blood	3/day*2 days	117	0.65	Status: EN
										Trend: D
*Aquila rapax* (Temminck, 1828)	Tawny Eagle	Tai	Mbeshi	Feathers	Treat chest pain.	Burn and inhale the smoke	15 minutes/day*3 days	108	0.60	Status: LC
										Trend: S
*Gallus domesticus* (Linnaeus, 1778)	chicken	Kuku	Ng’oko	Fat Egg white	Nasal congestion. Treat: dysentery	Rub the fat in the nasal Drink egg white	3/day*3 days Twice a day	145	0.81	Status: NA
										Trend: U
*Threskiornis aethiopicus* (Latham, 1790)	African Sacred Ibis	Nyangenyang e	Nzela	Blood	Treat: rheumatism	Drink flesh blood	1/2 cup/day*7 days	59	0.32	Status: LC
										Trend: D
*Ceryle rudis* (Linnaeus, 1778)	Pied Kingfisher	Ndobhelendo bhele		Fat	Treat: back pain	Massaged on the back	2/day*4 days	142	0.79	Status: NT
										Trend: D
*Dendropicos stierlingi* (Reichenow, 1901)	Stierling's Woodpecker	Fulubeji		Intestinal fecal content	Treat: diarrhea	mix hot water with fecal content	2 cup/day*3 days	45	0.25	Status: NT
										Trend: S
*Anas indica* (Linnaeus, 1778)	Duck	Bata	Mbata	Fat	Treat: Pneumonia, Chest pain	Wormed and massaged on the chest	3/day*3 days	92	0.51	Status: NA
										Trend: U
**Fish**										
*Mormyrus kannume* (Forsskal, 1758)	Elephant snout fish	Domodomo	Shironge	Whole organism	Treat: hookworms; removal poisonous	Burn, grind, mix with hot water	1 cup/day*3 days.	169	0.94	Status: LC
										Trend: D
*Lates niloticus* (Linnaeus, 1778)	Nile Perch	Sangara	Mbuta	Gills	Treat: abdominal cramp	Pound and mix with water	1 cup/day*7 days	85	0.47	Status: LC Trend: U
*Oreochromis variabilis* (Boulenger, 1906)	Victoria tilapia	Sato	Sato	Scales	Treat: cough	Burn and swallow the ashes	Regularly.	145	0.81	Status: CR
										Trend: D
*Octopus vulgaris* (Cuvier, 1797)	Common octopus	Pweza	Naghala	Tail	Treat: Urinary retention	Burn and swallow its ashes	2/day*3 days	25	0.13	Status: NA
										Trend: U
**Gastropod**										
*Snail* (O.F. Muller, 1774)	Achatina fulica	Konokono	Nonga	Shell	Treat: leg pain; make chain	Burn, grind, mix with water	Rub 2/day .*3 days	132	0.73	Status: NA,
										Trend: U
**Oligochaeta**										
*Lumbricus terrestris* (Linnaeus, 1778)	Earthworm	Mnyoo		Whole	Treat impotence	Dry; paste mix with hot water	2 spoon/day *7 days	99	0.55	Status: NA
										Trend: U

**LC** = Least Concern, **NT** = Near Threatened, **VU** = Vulnerable, **EN** = Endangered, **CR** = critically endangered, **NA** = Not Assessed, **I** = Increasing, **D** = Decreasing, **S** = Stable, **U** = Unknown, * = Times, **E** = English, **S** = Swahili.

**Figure 6:**
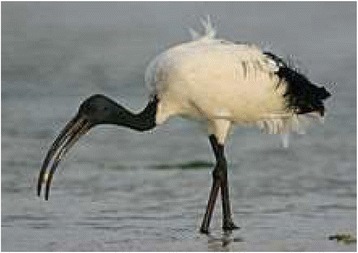
Threskiornis aethiopicus.

**Figure 7:**
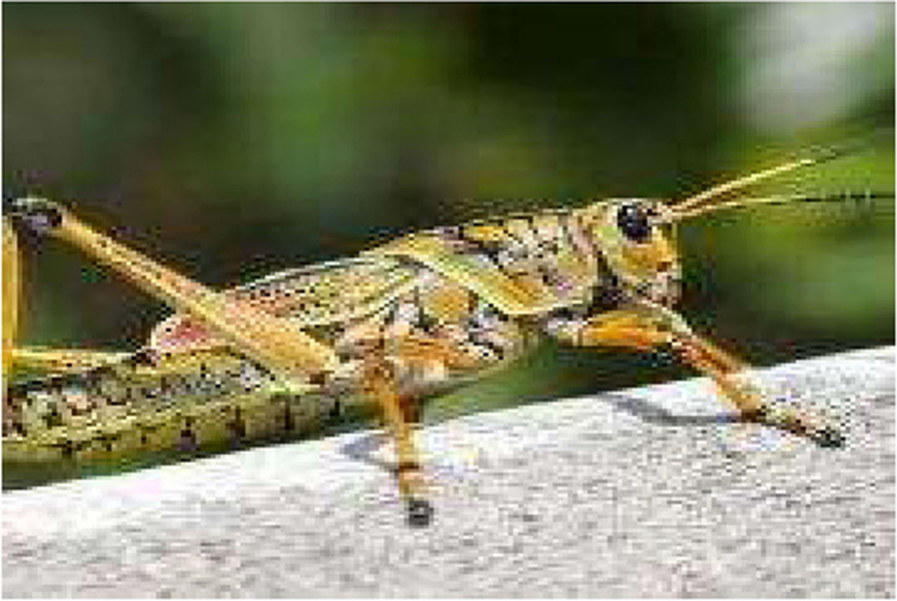
Butastur rufipennis.

**Figure 8:**
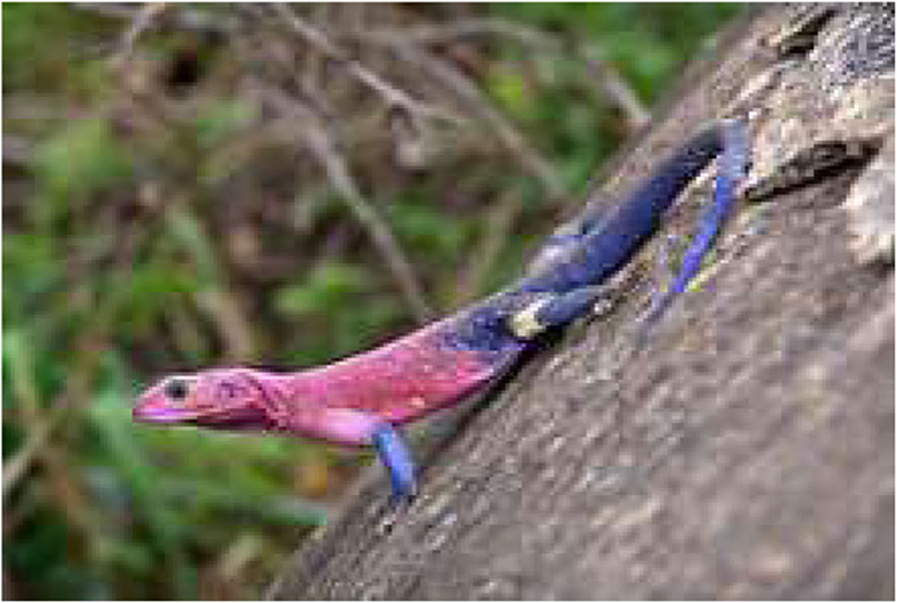
Agama Mwanzae.

**Figure 9:**
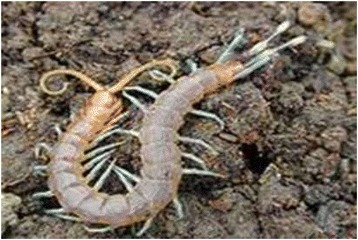
Trigoniulus corallines.

**Figure 10:**
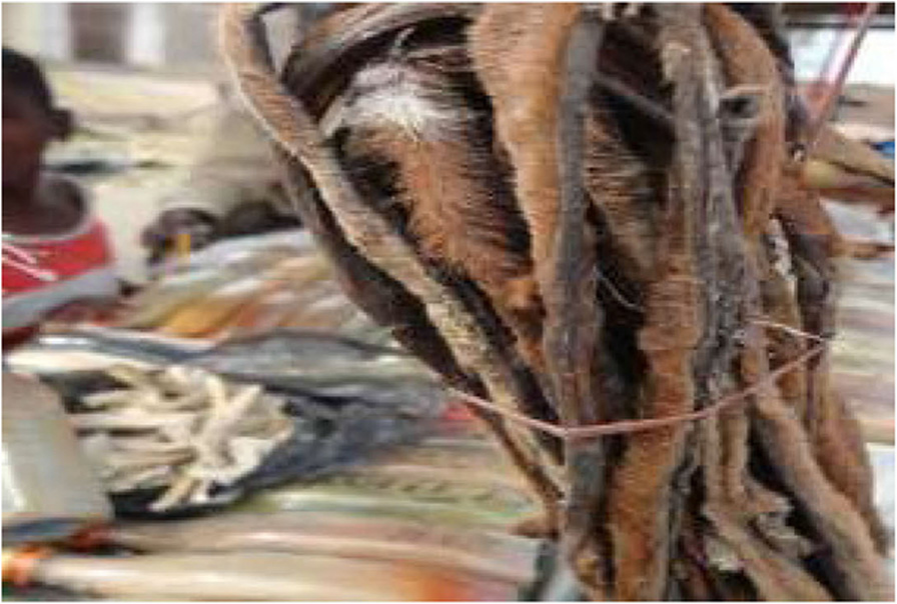
Skin of Panthera leo.

**Figure 11:**
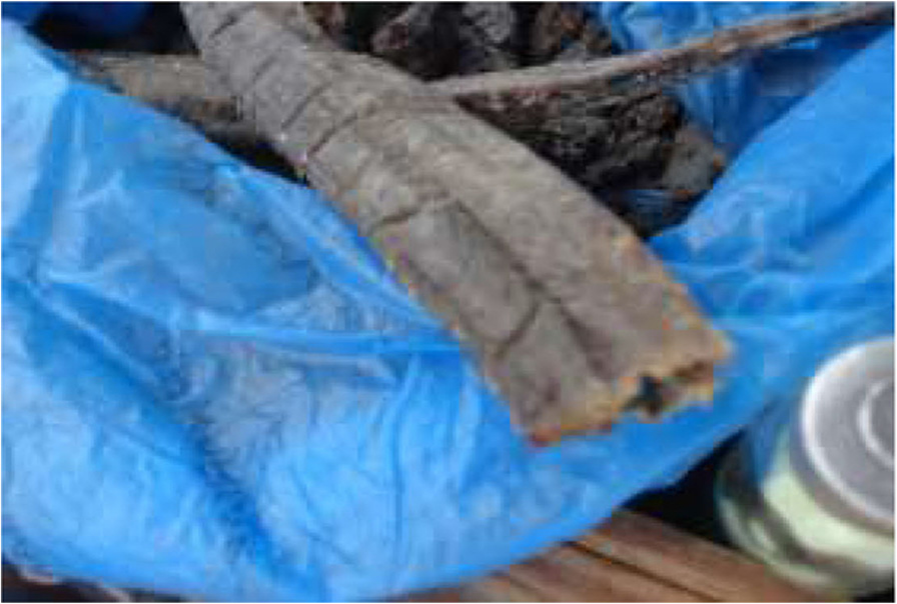
Dried Mormyrus kannume.

**Figure 12:**
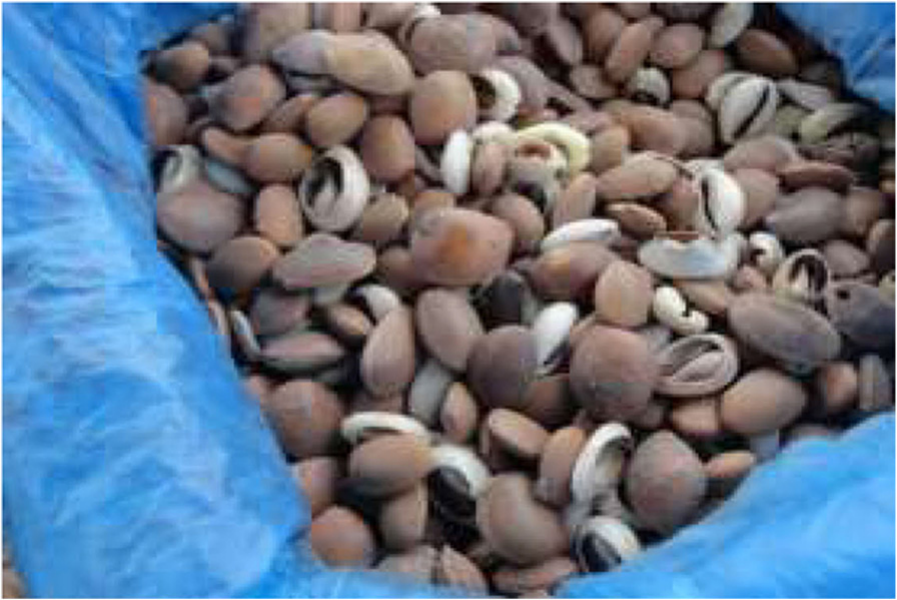
Achatina Fulica Shell.

**Figure 13:**
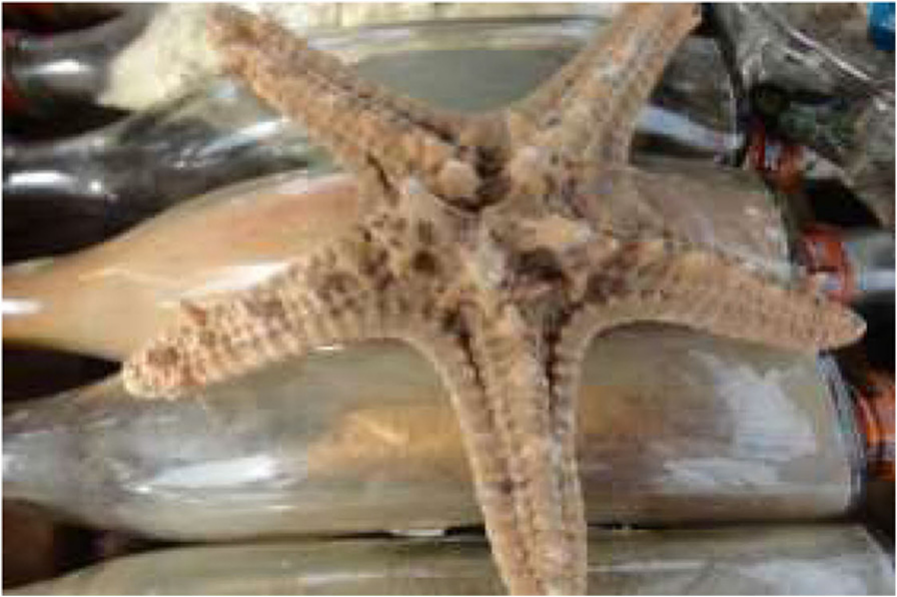
Dried Asterias sp.

The Table 1 shows that, Sukuma people of Busega district were using 42 animal species for the treatment of over 30 different kinds of illnesses. The animal species used as traditional medicine by these people comprise of seventeen mammals, seven birds, four reptiles, eight arthropods and two mollusks species. Highest number of animal belonged to mammalian taxonomic group (n = 17, 41%), birds (n = 7, 17%), reptiles (n = 4, 9.5%), fishes (n = 4, 9.5%) and arthropods (n = 8, 19%) respectively. Sukuma people use these animal and their products for the treatment of more than 30 types of different illnesses including asthma, paralysis, cough, fever, cold, STD, wound healing etc. These animals were used as whole or byproducts of these animals like milk, blood, organ, flesh, tooth, urine, honey, feather etc. for the treatment of various illnesses and used in the preparations of traditional medicine [Figures 6, 7, 8, 9, 10, 11, 12, 13].

Fidelity levels (FL) demonstrate the percentage of respondents claiming the use of a certain animals for curing of the illness. The uses of animals that are generally known by the Sukuma respondents have higher fidelity level is shown in Table 1.

Table: 1 also shows that cough, Tuberculosis, asthma, and other respiratory diseases are most frequently quoted disease among Sukuma people, as such, a number of traditional medicine are available for the treatment of such diseases, many animal byproducts were used like flesh of gazelle, horn of rhino, nail of mungos, and honey are some of them. Another important aspect of the present study that needs to be mentioned is that the Sukuma people also use some endangered, vulnerable and near threatened animal species as medicinal resources. A total of 42 identified animal species, of which 12 (28.57%) are included in the IUCN Red Data list [32]. It is important to mention here that species such as Tanzanian woolly bat, grey crowned crane, are listed as endangered while Black rhino and Victoria tilapia are listed as critically endangered and hippopotamus, African elephant, Simba (*Panthera leo*), Cobra (*Naja siamensis*) are listed as vulnerable in IUCN Red Data list. These tribal people have scarce knowledge, many irrational belief and myths associated with customs that cause harm to animal life. Thus these traditional medicine and animals byproducts should be tested for their appropriate medicinal components, if cited animal species among these people, byproducts of these animals, were used in the treatment of various illnesses.

Sukuma people also use one animal product with other animal products or plant derivatives to found indefensible, the people should be aware about the endangered and protected animal species and their importance in biodiversity. Consequently, the socio-ecological system has to be strengthened through sustainable management and conservation of biodiversity [33] [Table 2].

**Table 2:** **Conservation status of animal utilized in traditional medicine**

**IUCN red list category 2013**	**Frequency**	**Percent**
Least concern	20	47.62
Near threatened	04	9.52
Vulnerable	04	9.52
Endangered	02	4.76
Critically endangered	02	4.76

**Figure 14:**
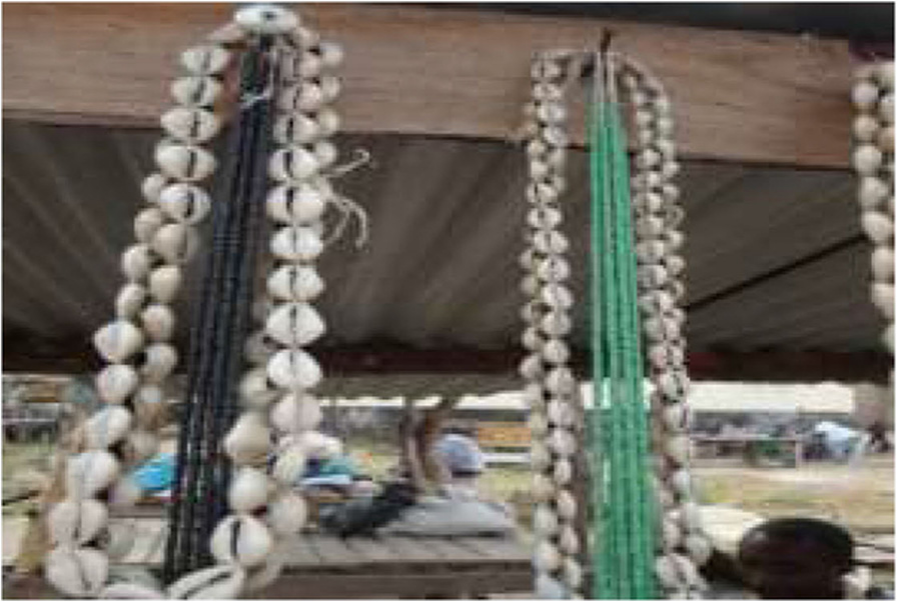
Different products obtained from animal resources among Sukuma Tribes.

**Figure 15:**
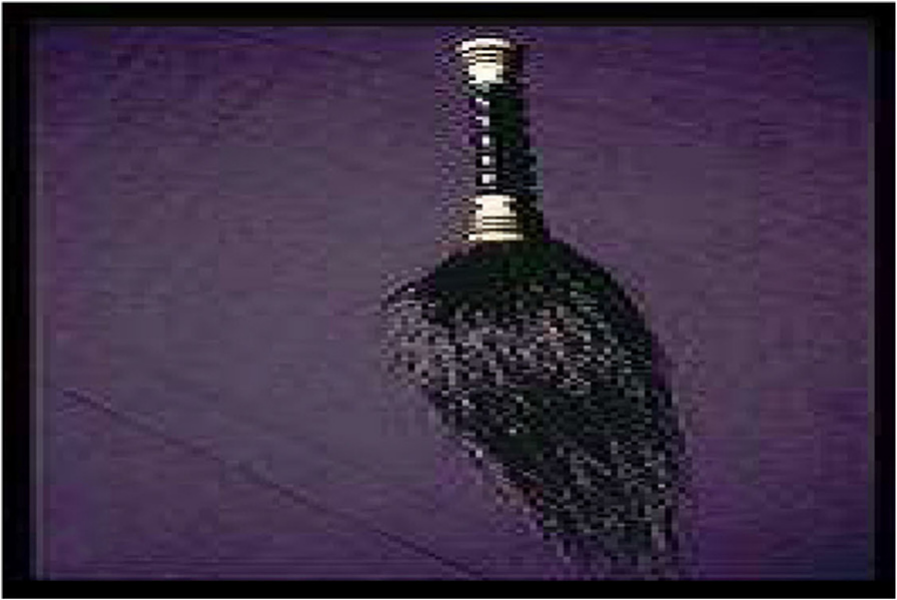
Different products obtained from animal resources among Sukuma Tribes.

**Figure 16:**
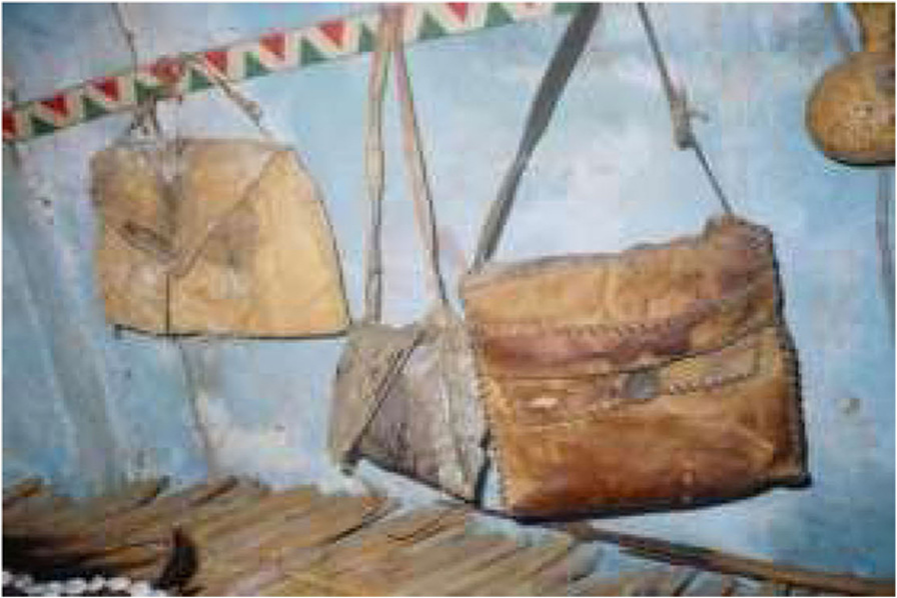
Different products obtained from animal resources among Sukuma Tribes.

**Figure 17:**
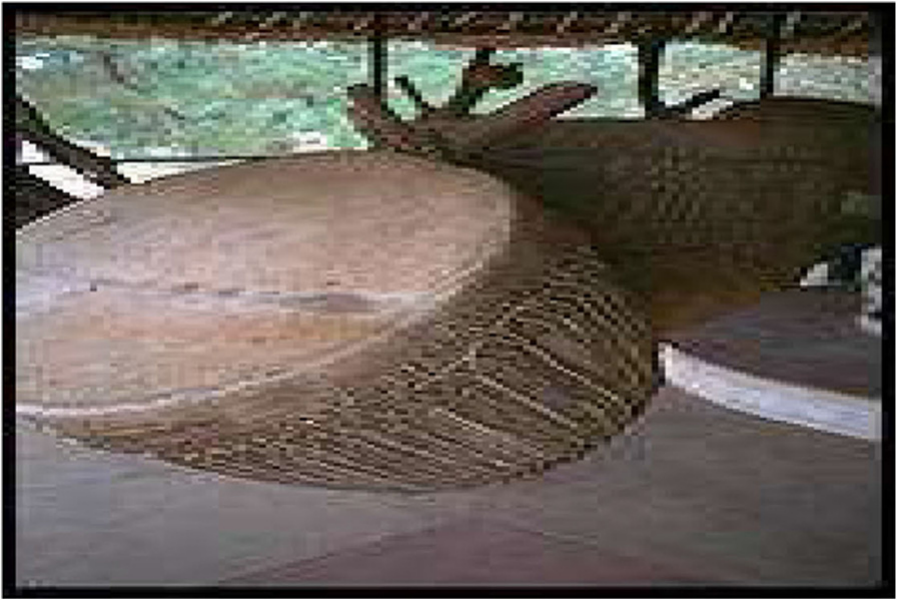
Different products obtained from animal resources among Sukuma Tribes.

Main threats of conservations in Tanzania includes overexploitation of natural resources due to poverty, rapid human population growths, weak wildlife policy and legislations, habitat alterations as well as inadequate funding. Poaching or illegal off take of wildlife resources has gone continuously regardless of wildlife conservation laws. However, traditional hunters in Tanzania have not been serious threat to wildlife. Wildlife populations are threatened by commercial poaching in which animal are used in bush meat trade and traditional medicine [34]. Despite medicinal purpose, Sukuma people also use animal resources for other purpose in their daily life. The Sukuma people use slough (molted skin of various animals) to decorate their traditional houses and this type of decoration are also reported in many other tribes living in other parts of Tanzania [Figures 14, 15, 16, 17].

## Conclusion

The current study shows that forty two animals were found to be used among Sukuma tribe of Busega district. Twelve animal species are officially considered as threatened species by IUCN red list (2012) were found among the set of faunistic resources prescribed as medicines at the time of this research. The latter author noted that Sukuma healers who are also diviners are more likely to use both wild and domesticated animals in their diagnoses. Moreover mammals, reptiles, birds, fish, and amphibians have been used in the field of traditional medicine for different purposes. However, mammals seem to be used much (40.50%) compare to other group among Sukuma tribe, followed by aves (16.7%). Amphibians are not commonly used in Sukuma society.

The present study also shows that the Sukuma people have very rich folklore and traditional knowledge in the utilization of different animal. So there is an urgent need to properly document to keep a record of the ethnomedicinal data of animal products and their medicinal uses. More studies are prerequisite for scientific validation to endorse medicinal value of such products and to include this knowledge in policies of conservation and management of animal resources. We hope that the present information will be helpful in further research in the field of ethnozoology, ethnopharmacology and biodiversity conservation viewpoint.
